# Supplementary material for: Breakpoint–chiasma interference in pericentric inversion heterokaryotypes
Source: Genetics. 2025 May 6;230(2):iyaf070. doi: 10.1093/genetics/iyaf070 (PMC12135173; doi:10.1093/genetics/iyaf070)
Supplement: iyaf070_Supplementary_Data [file iyaf070_supplementary_data.pdf]

Table S1 Expected and observed counts for inversion 165. The observed data is sourced from Roberts (1967).

Pattern	Observed	Expected H_0	Expected H_1	Expected H_2
0000000	1259	1263.3	1261.2	1265.44
0000001	248	240.3	243.2	243.4
0000010	585	558.7	569.1	567.6
0000011	43	53.7	51.7	50.7
0000100	274	281.8	281.4	279.3
0000101	55	49.8	50.5	50.7
0000110	62	62.0	58.2	58.5
0000111	3	4.8	4.0	3.9
0001000	157	164.7	158.8	157.22
0001001	34	31.4	30.9	30.8
0001010	54	57.2	57.5	58.8
0001011	3	5.0	4.8	4.8
0001100	2	9.2	10.5	10.7
0001101	4	1.6	1.8	2.0
0001110	6	1.8	1.9	2.0
0001111	1	0.1	0.1	0.1
0010000	19	24.5	22.0	21.5
0010001	5	4.7	4.3	4.2
0010010	9	9.5	9.5	9.6
0010100	3	2.3	3.6	3.7
0010101	1	0.4	0.6	0.7
0010110	1	0.4	0.7	0.8
0011100	1	0.0	0.1	0.1
0110000	6	7.8	7.8	7.8
0110001	2	1.5	1.5	1.5
0110010	2	3.5	3.5	3.5
0111000	1	1.0	1.0	1.0
0111010	1	0.4	0.4	0.4
0111100	1	0.0	0.1	0.1
0100000	5	0.2	0.2	0.2
1010000	2	2.8	2.7	2.7
1010001	2	0.5	0.5	0.5
1010100	1	0.6	0.6	0.6
1011000	1	0.3	0.3	0.3
1000000	10	8.3	8.3	8.2
1000100	1	1.8	1.8	1.8
1001000	2	1.1	1.0	1.0
1001001	2	0.2	0.2	0.2
1001010	2	0.4	0.4	0.4
1001100	1	0.1	0.1	0.1
1100000	3	1.9	2.0	2.0
1100110	1	0.1	0.1	0.1

Table S2 Expected and observed counts for inversion 269. The observed data is sourced from Roberts (1967).

Pattern	Observed	Expected H_0	Expected H_1	Expected H_2
0000000	2961	2998.3	2990.3	2989.7
0011000	21	18.4	18.5	17.7
0010100	26	23.2	23.5	23.3
0001100	24	16.3	15.8	16.8
0010010	18	13.0	12.5	13.5
0001010	18	17.2	17.2	16.9
0000110	5	2.4	2.4	2.0
0010000	28	34.4	30.5	31.3
0001001	3	1.6	1.6	1.5
0001000	2	0.2	0.2	0.2
0000100	4	0.3	0.2	0.2
1000000	1293	1248.6	1262.2	1261.8
1000001	3	1.0	1.0	1.0
1011000	3	7.6	7.8	7.5
1010100	2	9.7	9.9	9.8
1001100	2	6.8	6.7	7.1
1010010	2	5.4	5.3	5.7
1001010	5	7.2	7.2	7.1
1001000	5	10.4	11.4	10.6
1001001	2	0.7	0.7	0.6
1000100	2	0.1	0.1	0.1
1000010	1	0.1	0.1	0.1
0100000	750	730.4	734.0	736.2
0100001	1	0.6	0.6	0.6
0111000	3	4.5	4.5	4.4
0110100	4	5.6	5.8	5.7
0101100	3	4.0	3.9	4.1
0110010	1	3.1	3.1	3.3
0101010	2	4.2	4.2	4.2
0100010	2	0.004	0.001	0.01
1100000	128	141.6	133.5	132.5
1111000	1	0.9	0.8	0.8
1110010	1	0.6	0.6	0.6
1101010	1	0.8	0.8	0.7
1101000	2	0.001	0.003	0.002
1100100	1	0.001	0.004	0.003

Table S3 Expected and observed counts for inversion 190. The observed data is sourced from Roberts (1967).

Pattern	Observed	Expected H_0	Expected H_1	Expected H_2
0000000	1939	1958.7	1945.7	1947.3
0000001	397	388.3	402.1	401.3
0000010	497	467.9	480.1	479.1
0000011	3	10.7	4.5	4.6
0000100	22	22.4	21.7	21.3
0000110	2	0.1	0.4	0.
0100000	17	18.8	17.6	17.5
0100001	3	3.7	3.6	3.6
0100010	4	4.5	4.3	4.3
0100100	1	0.2	0.2	0.2
1000000	242	250.9	244.9	244.9
1000001	64	49.7	50.6	50.5
1000010	47	59.9	60.4	60.2
1000100	2	2.9	2.7	2.7
1100000	2	0.01	0.1	0.1

Table S4 Expected and observed sterilities of pericentric inversions on chromosome 3 of *D. melanogaster*. The observed data is sourced from Coyne et al. (1993).

Inversion	I	ρ	Observed sterility	Expected H_0	Expected H_1	Expected H_2
273	0.19	0.63	-0.0702	0.046	0.0029	0.0029
238	0.11	0	-0.0518	0.027	0.0019	0.0019
281	0.24	0.8	-0.0372	0.060	0.013	0.013
LD31	0.18	0.99	-0.0338	0.046	0.0108	0.108
265	0.144	0.877	-0.0319	0.036	0.0031	0.0031
224	0.085	0.94	-0.0300	0.021	0.00057	0.00057
275	0.201	0.50	-0.0263	0.050	0.0026	0.0026
277	0.242	0.991	-0.0258	0.061	0.027	0.027
280	0.101	0.99	-0.0258	0.025	0.0013	0.0013
C190	0.2	0.4	-0.0057	0.049	0.0031	0.0031
234	0.21	0.66	-0.0051	0.051	0.0048	0.0048
260	0.281	0.99	0.0248	0.071	0.043	0.043
LD12	0.29	0.99	0.0251	0.068	0.0386	0.0386
252	0.354	0.79	0.0469	0.086	0.043	0.043
270	0.3	0.93	0.0512	0.075	0.043	0.043
Sep	0.27	0.93	0.0672	0.0672	0.0306	0.0306
259	0.44	0.93	0.0758	0.11	0.1185	0.1185
267	0.221	0.99	0.0895	0.056	0.0204	0.0204
111	0.835	0.467	0.1009	0.19	0.218	0.218
278	0.48	0.98	0.1072	0.12	0.16	0.16
268	0.40	0.99	0.1087	0.10	0.105	0.105
250	0.57	0.79	0.1108	0.14	0.148	0.148
279	0.58	0.81	0.1390	0.14	0.162	0.162
272	0.485	0.9381	0.1622	0.12	0.15	0.15
282	0.221	0.99	0.1674	0.056	0.021	0.021
257	0.359	0	0.1868	0.09	0.085	0.085
C269	0.52	0	0.1996	0.13	0.1974	0.1974
LD3	0.471	0.99	0.1994	0.12	0.161	0.161
208	0.5	0.94	0.2086	0.12	0.161	0.161
271	0.471	0.99	0.2762	0.12	0.161	0.161
